# DRG grouping by machine learning: from expert-oriented to data-based method

**DOI:** 10.1186/s12911-021-01676-7

**Published:** 2021-11-09

**Authors:** Xiaoting Liu, Chenhao Fang, Chao Wu, Jianxing Yu, Qi Zhao

**Affiliations:** 1 School of Public Affairs, Zhejiang University, Zijingang Campus, Hangzhou, 310058 Zhejiang Province China; 2 Centre of Social Welfare and Governance, Zhejiang University, Hangzhou, China; 3 College of Control Science and Engineering, Zhejiang University, Hangzhou, China; 4 School of Public Administration, Zhejiang Gongshang University, Hangzhou, China

**Keywords:** Diagnosis-related groups (DRGs), Grouping, Machine learning, China, Healthcare

## Abstract

**Background:**

Diagnosis-related groups (DRGs) are a payment system that could effectively solve the problem of excessive increases in healthcare costs which are applied as a principal measure in the healthcare reform in China. However, expert-oriented DRG grouping is a black box with the drawbacks of upcoding and high cost.

**Methods:**

This study proposes a method of data-based grouping, designed and updated by machine learning algorithms, which could be trained by real cases, or even simulated cases. It inherits the decision-making rules from the expert-oriented grouping and improves performance by incorporating continuous updates at low cost. Five typical classification algorithms were assessed and some suggestions were made for algorithm choice. The kappa coefficients were reported to evaluate the performance of grouping.

**Results:**

Based on tenfold cross-validation, experiments showed that data-based grouping had a similar classification performance to the expert-oriented grouping when choosing suitable algorithms. The groupings trained by simulated cases had less accuracy when they were tested by the real cases rather than simulated cases, but the kappa coefficients of the best model were still higher than 0.6. When the grouping was tested in a new DRGs system, the average kappa coefficients were significantly improved from 0.1534 to 0.6435 by the update; and with enough computation resources, the update process could be completed in a very short time.

**Conclusions:**

As a new potential option, the data-based grouping meets the requirements of the DRGs system and has the advantages of high transparency and low cost in the design and update process.

## Introduction

In the most recent healthcare reform, China has made substantial progress in improving equal access to care and enhancing financial protection. However, gaps remain in efficiency in the delivery and control of health expenditures [1]. With the enhancement and standardisation of medical information systems and clinical pathways, the Chinese government has paid closer attention to payment reform and enhanced supervision of the quality of medical care in the new round of healthcare reform, hoping to curb soaring medical expenditures [2, 3]. One of the core measures is provider payment reform, in which diagnosis-related groups (DRGs) payment is perceived as a valuable alternative to the conventional fee-for-service (FFS) payment method. In 2009, the Chinese government announced the initiation of the prospective DRG-based payment reform. Until 2016, two national DRG groupings, CN-DRGs and C-DRGs, were developed and tested in Sanming, Shenzhen and Karamay, and nearly twenty of the thirty-two provinces in mainland China implemented the simplified DRGs.

Originating from Yale University and first implemented in the United States in 1983 [4], DRGs is a payment system that can gather patients with similar clinical symptoms and similar resource consumption patterns into the same group. The medical expenses that patients and medical insurance need to pay are only related to the results of grouping [5, 6]. In the DRGs system, excessive drugs and treatment provided by hospitals will not be paid for, which improves healthcare quality and stabilises costs [7].

Aiming at allowing for more ‘outside’ control on hospital expenditure, several pieces of common grouping software have been developed to standardise and facilitate hospital payments in China. However, as in many other countries, the basic DRGs structure has undergone numerous revisions since its creation, leading to a less stable, more complex, and often confusing process [8]. The grouping, an exhaustive patient case classification system, is the core design characteristic of a DRG-based payment system [9]. Treatment trajectory encoding information about a patient and their clinical treatment is put through a large formal decision tree—the grouping, which consists of thousands of decision rules, each evaluating to either true or false. By traversing these decision rules, a care product is defined and determined [10]. As the grouping is a black box, the decision-making rules of which are not disclosed to the public, its algorithmic nature makes reimbursing a highly technical endeavour. Due to the complexity and lack of transparency of the grouping software, on the one hand, it might spark a public debate about whether providers and professionals might use the system to further their interest [10]; on the other hand, as clinicians have stated, the grouping software has rendered the payment process too complex and error-sensitive, leading to remuneration errors and subsequent loss of hospital income [11].

Moreover, with the purpose of cost control, DRGs payment is usually supported by the mechanism of Global Budget and a maximum growth percentage for hospital care in the government’s pilot practices of DRGs [12]. Thus, in the special context of healthcare reform in China, the grouping software of DRGs is embedded within political concerns and measurements. Though it is necessary to keep the care code correct by updating it, most of the pilot reforms have a tendency towards scientism, elitism, and mysticism, leading to bureaucratic powers playing a leading role in the processes of grouping, pricing, and quality assurance of DRGs [13], as stated by local researchers. As a result, the effects of DRGs payments on healthcare were mixed. Empirical evidence demonstrated that DRGs payment may mildly improve the efficiency but impair the quality and equity of healthcare, and may yield upcoding of medical records [14].

**Fig.1:**
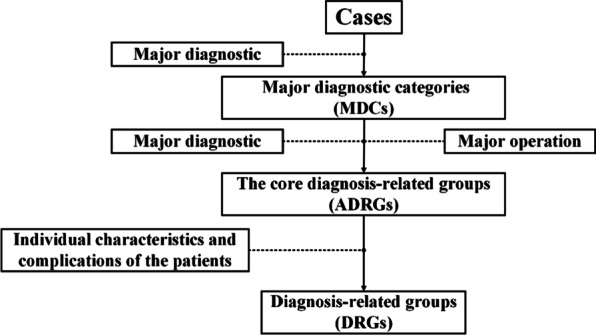
The CHS-DRG grouping

However, it is challenging for expert-oriented grouping to solve these problems. For example, in October 2019, the National Healthcare Security Administration in China officially promulgated “the technical specification of China Healthcare Security Diagnosis Related Groups (CHS-DRG)” [15]. As shown in Fig. 1, this specification proposed a traditional DRG grouping, which divided the grouping into two parts. One part contains decision-making rules based on clinical similarities in cases, the other part contains decision-making rules based on individual similarities in patients including the length of stay, age, complications, etc. They are connected by the core diagnosis-related groups which were named ADRGs.

**Fig.2:**
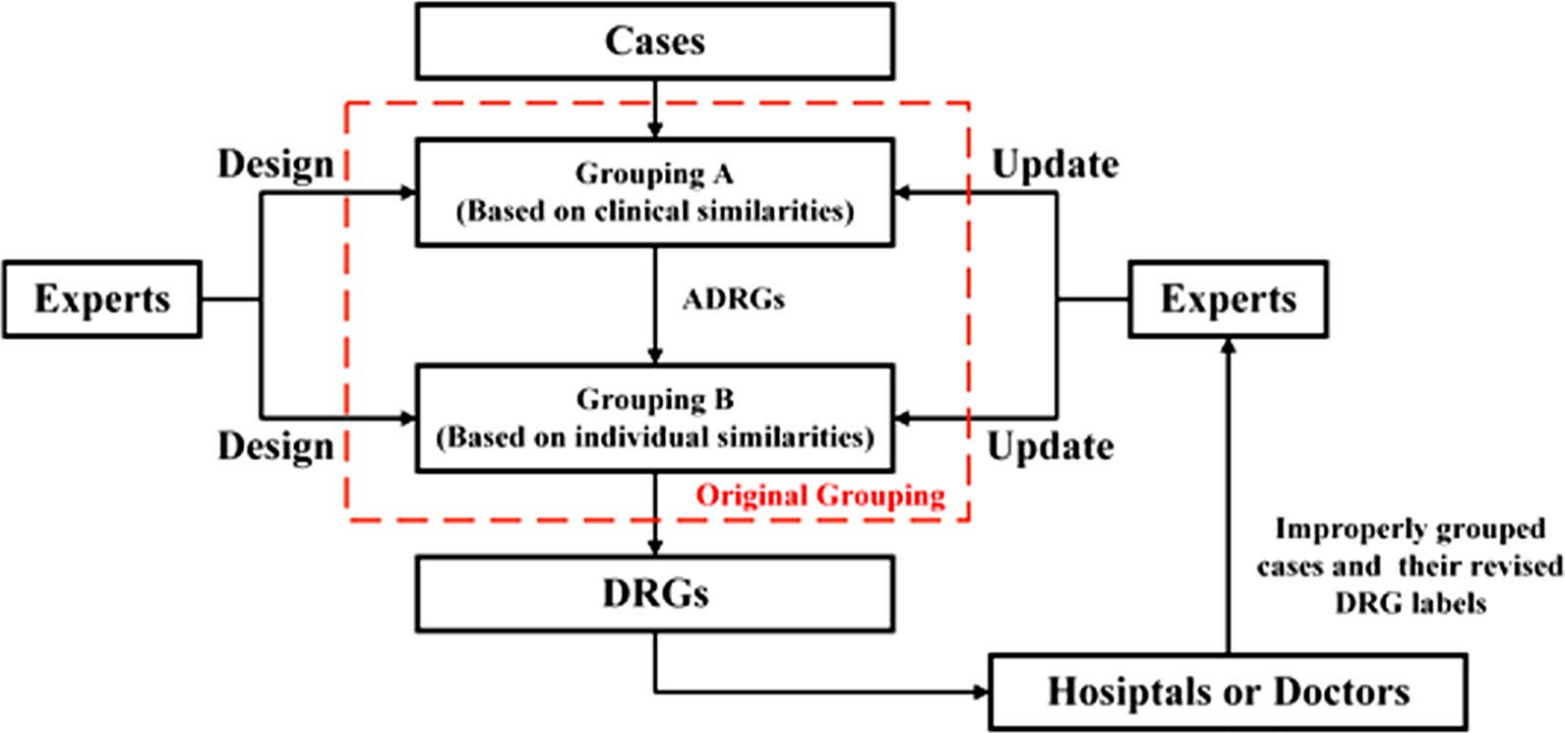
Execution flow diagram of expert-oriented grouping

Figure 2 employs a flow diagram to show how a traditional expert-oriented DRG grouping works. As shown in the figure, both the design and update of the grouping rely heavily on the decisions of experts. When doctors get DRG grouping software, because it is a black box that could not give any effective suggestions on improvement. What is more, it is completely impossible to provide localised grouping for different hospitals, when the design or updating of any grouping requires the input of many professional medical associations, specialist experts and consultants via a series of scientific, and rigorous procedures such as committees, expert hearings and consultations [16, 17].

Currently, machine learning provides many new tools for groupings design. There is a growing body of literature discussing machine learning and various algorithms for DRG grouping and resource allocation [18–20]. These studies focus on applying machine learning to improve the accuracy of DRG groupings and proving that machine learning could effectively assist in hospital management and resource allocation. However, they do not discuss further whether machine learning provides a new DRG grouping pattern which is data-based to eliminate unnecessary human intervention and restriction.

The data-based structure is an attractive DRG grouping design concept because it could avoid easily the drawbacks of the expert-oriented structure. Both providers and consumers could access, modify and validate decision-making rules in the data-based groupings through exposed machine-learning methods, and machine-learning methods also endow groupings with strong growth capacity with the support of sufficient computing resources. In this research, we propose such a data-based grouping and try to explore a data-based grouping built by machine learning, which could replace the current expert-oriented grouping with higher transparency and simpler design processes.

## Available/generated data

Since real cases involve personal privacy, it is quite difficult for researchers to obtain enough real cases directly to design groupings. It is, however, much easier to get the feature distribution of real cases through some statistical reports from the governments. Therefore, we generated 2,000,000 simulated cases based on the feature distribution of real cases in Zhejiang province in 2018, which was provided by the Health Commission of Zhejiang Province for groupings training. What is more, we also got 1,062 real cases from the Dongyang People’s Hospital from November 2019 to December 2019 to verify the performance of groupings. All cases consist of features as shown in Table 1. A simple description of patient characteristics of the real cases is summarised in Table 2.

**Table 1:** The features contained in cases

Case sample variables
Major diagnosis (ICD-10)
Major operation (ICD-9)
Secondary diagnosis (ICD-10)
Age
Sex
Length of stay
Neonatal days
Birth weight of the new-born
Weight of new-born at the time of diagnosis
Total treatment expense

**Table 2:** Patient characteristics of the real cases

Variable	Sample size
*Gender*	
Male	456
Female	606
*Age (years)*	
≤ 20	142
21–40	107
41–60	330
> 60	483
*Length of stay (days)*	
≤ 5	441
6–15	512
> 15	109
*Major diagnosis distribution*	
Circulatory disease and dysfunction	42
Neurological disease and dysfunction	430
Musculoskeletal disease and dysfunction	245
Diseases and dysfunction of the female reproductive system	219
Neonatal and other perinatal neonatal diseases	127

In any case, categorical variables are encoded numerically to the positive number, such as major diagnosis, sex, etc. The encoded data can adapt to scikit-learn while is a widely used machine learning toolkit in Python and is normalized by Min–Max scaling. There is no feature information loss in the simulated data and the real data, and the data generation algorithm and the hospital information system guarantee their integrity. However, to make sure that each case can be converted to a data format that the model can accept, we need to populate some features according to the following rules. For patients who are not infants, there are no features of neonatal days, birth weight of the new-born, and birth weight of the new-born, and not everyone normally has a major operation. We use the value of − 1 to make up them because this value is impossible to appear in normal neonatal cases. What’s more, the number of secondary diagnoses varies greatly from case to case which is up to five. To balance the calculation complexity and simulation authenticity, both the simulated data and real data have three secondary diagnoses which are arranged from small to large according to the encoding. If the number of secondary diseases in the original data is less than three, the missing features will be made up with the value of − 1 as well.

The simulated cases and real cases obtained the ADRG labels and DRG labels by being imported into the CN-DRGs-B grouping which is executed through software. The real cases have another set of ADRG labels and DRG labels, which were provided by the local healthcare security administration. In the experiment, these local labels were seen as the revised labels based on the actual local medical cost. Finally, three datasets were set up for different validation goals. The size and a brief description of the datasets are shown in Table 3.

**Table 3:** Description of the validation datasets

Dataset	Description	Sample size
A	Simulated cases with labels generated by the CN-DRGs-B grouping	2,000,000
B	Real cases with labels generated by the CN-DRGs-B grouping	1062
C	Real cases with local labels	1062

## Methods

### Machine-learning models

**Fig. 3:**
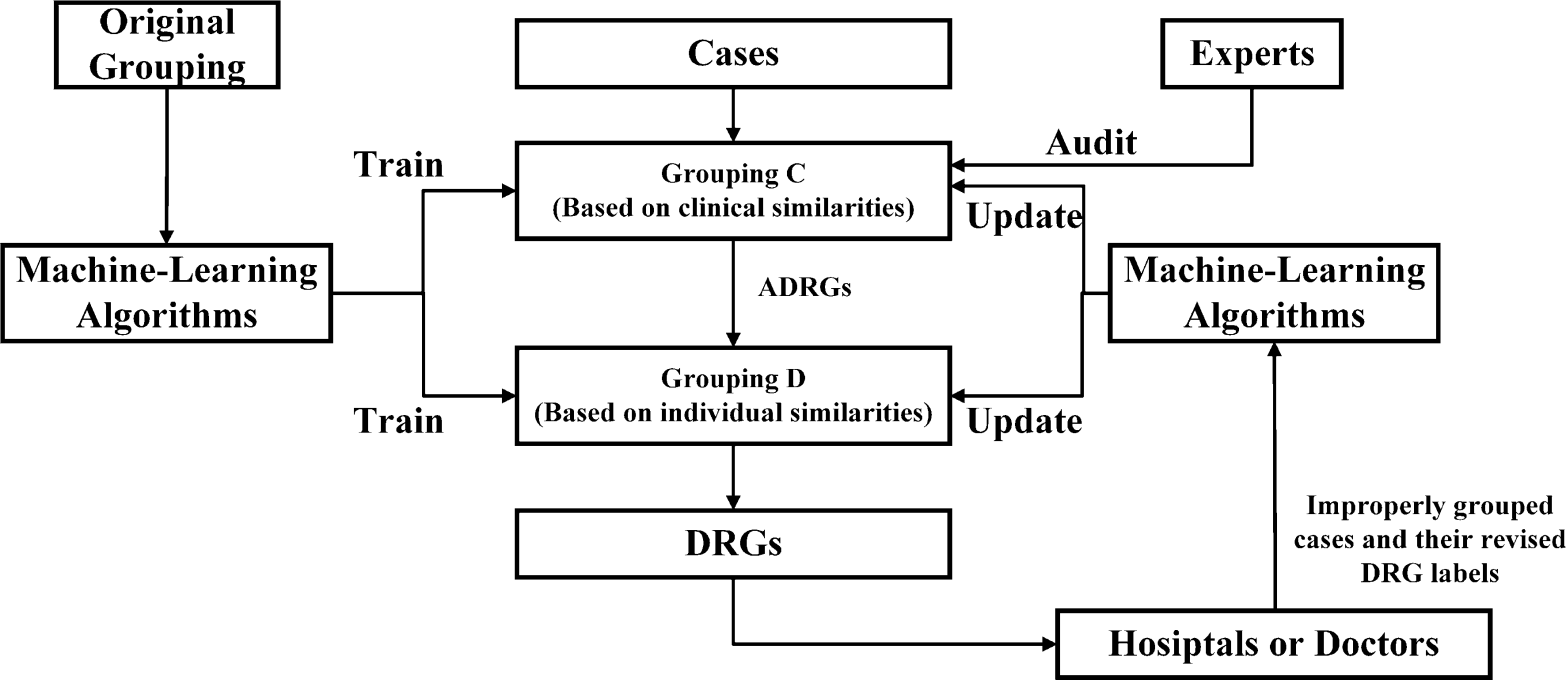
Flow diagram of the execution of data-based grouping

In the optimised grouping, we use machine-learning algorithms to replace all the non-professional work of experts and propose a data-based grouping. Its execution flow is shown in Fig. 3. Taking advantage of real or simulated cases labelled by existing grouping, the new grouping could be designed by training. Then, the grouping is updated with respect to improperly grouped cases and their revised labels. The design and update are implemented by machine-learning algorithms rather than experts’ evaluation. Decision-making rules based on clinical similarities are open to experts and users. Experts still audit these rules according to their professional knowledge. Although the rules based on individual similarities may still be a black box, upcoding could be avoided because rules are invisible to both the designer and the user and are entirely determined by the data when the design method is open.

**Fig. 4:**
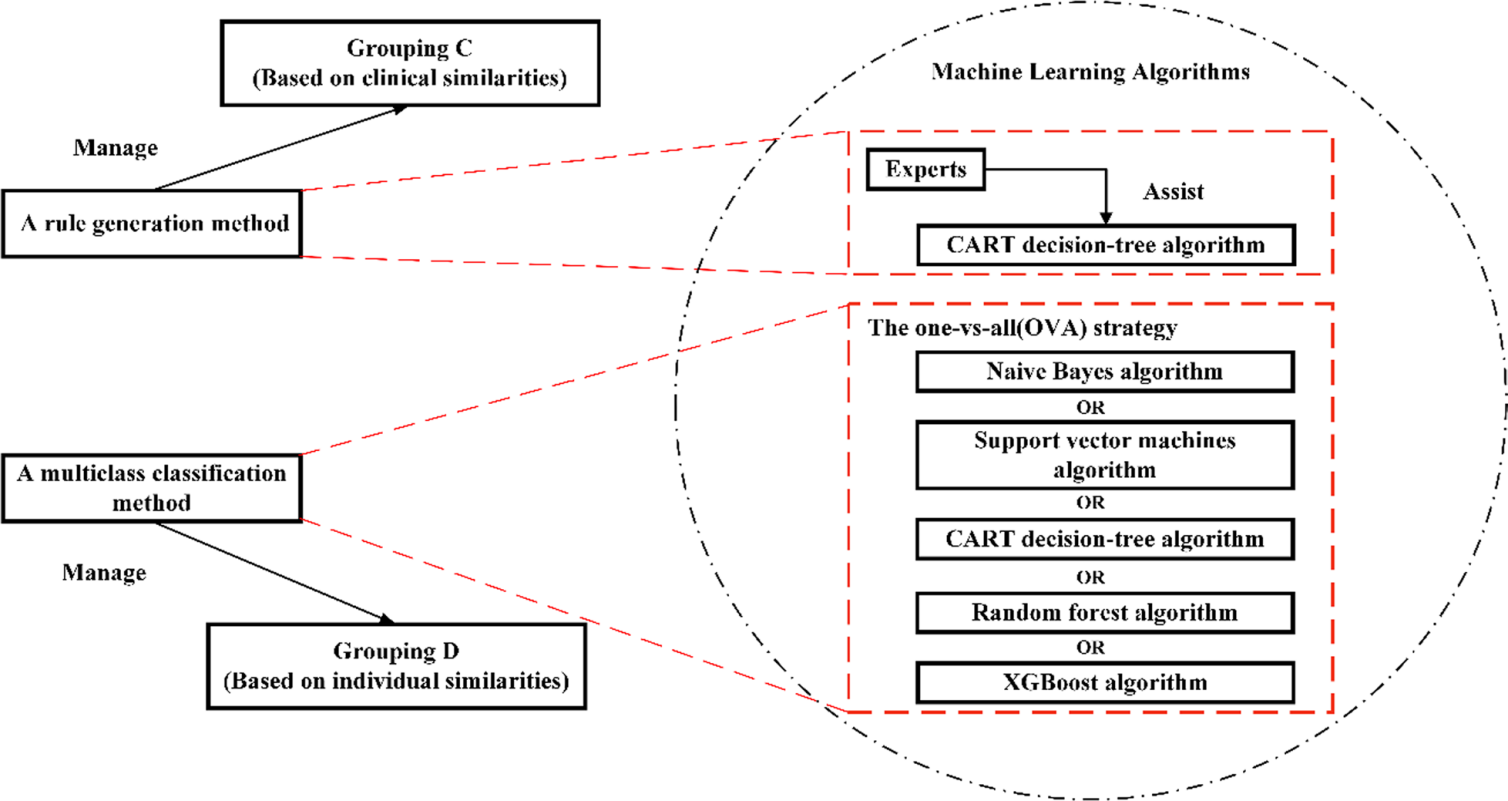
The framework of machine learning algorithms

As shown in Fig. 4, machine-learning algorithms are organised by a rule generation method and a multiclass classification method. They are applied to manage the part based on clinical similarities and the part based on individual similarities respectively. The details of both methods are described in the following sections.

### A rule generation method

The rule generation method needs to provide experts and users with a decision-making rules model which is easy to search and modify. The tree structure is an appropriate choice, which gives a compact intuitively interpretable representation of the statistical model. Trained by cases that only have the features of the major diagnosis and the major operation, a binary decision tree can be built by the CART algorithm [21]. The CART algorithm is an effective means to create conjunctive rules [22], which uses the Gini index to select partition attributes. The Gini index represents the purity of the dataset, so each node selects the rule that can minimise the Gini index of the divided dataset. The tree will grow until either the homogeneity of the nodes cannot be improved significantly or additional stopping criteria are met.

**Fig.5:**
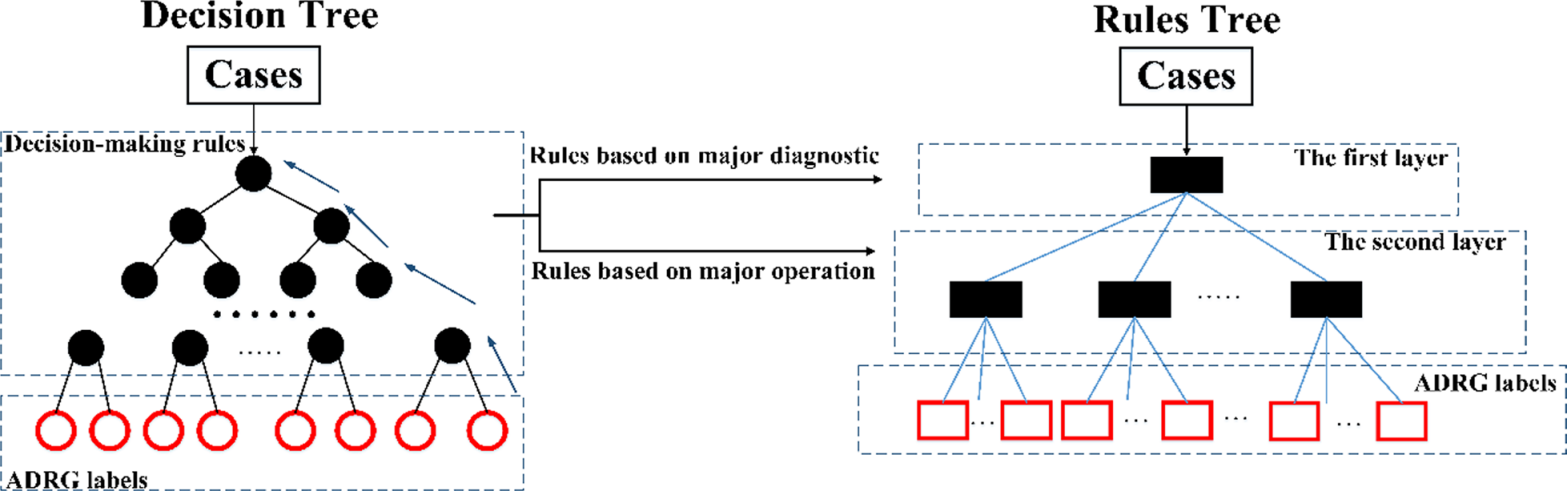
The process of generating a rules tree from a decision tree

**Fig.6:**
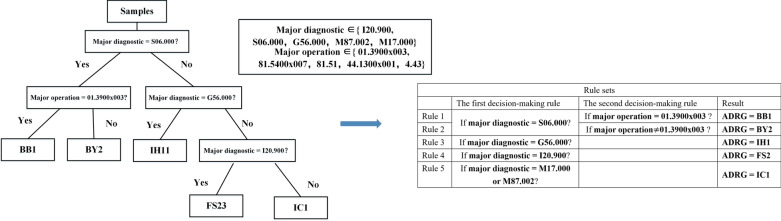
A specific example of the rule tree creation process

Splitting nodes are called internal nodes, and nodes without successors are termed terminal nodes. In the decision tree built by cases, every internal node has a decision-making rule and every terminal node can be interpreted as an ADRG label. Trees constructed in the CART algorithm tend to have too many internal nodes and layers for a classifier applicable to all DRGs, although it depended on a number of factors, such as the number of branches of each node. To solve this problem, the tree should be reconstructed and compressed since all decision-making rules have only two features. As shown in Fig. 5, the decision tree can be exported as a two-layer rules tree by tracing back from each terminal node to the top node and combining node rules with the same feature and same ADRG label, which is easy for computers. In the rules tree, the internal node in the first layer contains rules which are only based on the major diagnosis, and the internal nodes in the second layer contain rules which are only based on the major operation. Every internal node has several successors and every terminal node represents a different ADRG label. In order to facilitate readers' understanding, we will give a specific example to illustrate the rule tree creation process. Since different samples will not affect the creation process, we have selected a small number of samples for easy presentation in the article. Their details are shown in the following Table 4 and the creation process is shown in Fig. 6. The doctor injects six kinds of data shown in Table 4 into the model. The decision tree algorithm will continuously divide the data set until there is only one ADRG in each data set. At this time, each ADRG rule has at most three decision-making rules, which can be obtained by backtracking the nodes of the binary tree. The computer calculates the set of major diagnostic and major operations in this data set and splits both sets with the decision-making rules corresponding to each ARDG to obtain the final rules shown on the right side of Fig. 6. This example shows that the algorithm can guarantee that no matter how many kinds of data, only as many rules will be output as the number of ADRG types and each rule contains at most two decision-making rules. In practice, there are often thousands of data types, and this algorithm can effectively and quickly generate concise and accurate classification rules for doctors and researchers.

**Table 4:** Sample characteristics of the example

Major diagnostic (ICD-10)	Major operation (ICD-9)	ADRG label	Sample size
S06.000	01.3900 × 003	BB1	15
S06.000		BY2	34
M17.000	81.5400 × 007	IC1	11
M87.002	81.51	IC1	9
I20.900	44.1300 × 001	FS1	28
G56.000	4.43	IH1	5

In the update process, we generate a candidate rules tree by revised cases or new cases. The terminal nodes of the candidate rule tree only include the ADRG labels which have been changed. Experts can easily update the grouping by comparing the conflicting rules between the candidate rules tree and the original rules tree, and judging whether to modify them.

### A multiclass classification method

Dividing cases from ADRGs into DRGs is a complex multiclass classification problem involving several case features. Traditional decision-making rules cannot have both low design costs and accurate classification results in solving this problem, while there are many machine-learning algorithms employed in developing high-performance classification models. What’s more, in order to improve the multiclass classification capability of each method and reduce the workload of model updating, all algorithms work with the one-vs-all (OVA) strategy.

**Fig. 7:**
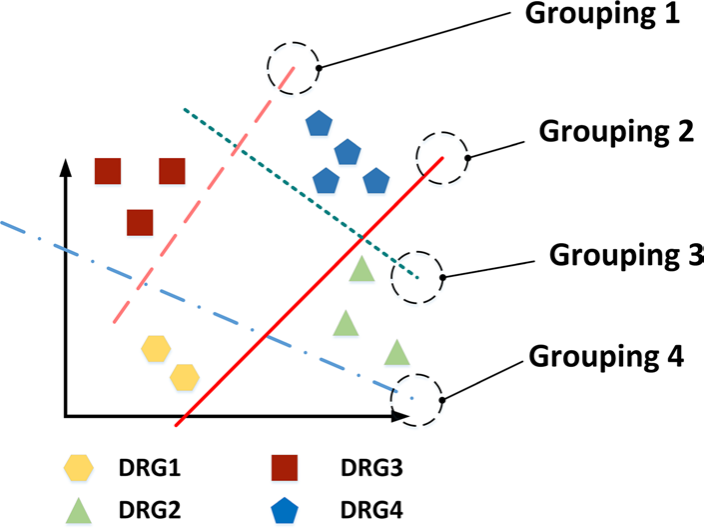
The one-vs-all (OVA) strategy

The OVA strategy, as shown in Fig. 7, consists of fitting one grouping per class. For each grouping, the class is fitted against all the other classes. Adopting this strategy will not reduce the classification accuracy [23], and can ensure that the grouping still has good interpretability. Since in this part the cases are classified from ADRGs into DRGs, and each ADRG contains no more than ten DRGs, so when training the model, the amount of training data for each DRG class is relatively balanced. If an ADRG contains dozens of DRGs as the grouping is continuously updated and expanded, we can choose an appropriate grouping to divide this large ADRG into two parts, and then continue to implement the OVA strategy.

In the update process, we first record which DRGs in the new training data have been changed, and then we just need to retrain their corresponding groupings. This method avoids retraining all groupings for each update, reduces the cost of computations resources, and makes updating easier.

### Model selection

In the multiclass classification method, many machine learning algorithms can be chosen. We describe and access five typical classification algorithms in this paper: Naive Bayes, Support Vector Machines (SVM), Classification and Regression Trees (CART), Extreme Gradient Boosting (XGBoost), and random forest, which is a reference for users to select algorithms. The CART decision-tree algorithm has been described in the previous section.

### Naive Bayes algorithm

Naive Bayes algorithm was designed with the assumption of conditional independence between every pair of features given the value of the class variable called class conditional independence [24]. Given class variable $y$ and dependent feature vector ${x}_{1}$ to ${x}_{n}$, in Naive Bayes algorithm, the posterior probability of a sample can be expressed as follows:

1 $$P(y\mid {x}_{1},\dots ,{x}_{n})=\frac{P(y)\prod_{i=1}^{n}P({x}_{i}\mid y)}{P({x}_{1},\dots ,{x}_{n})}$$

The sample will be classified into the class with the highest posterior probability and in this paper, the likelihood of the features is assumed to be Gaussian.

### Support vector machines (SVM) algorithm

The Support vector machines (SVM) algorithm was proposed by Cortes and Vapnik [25] and quickly became a mainstream technology in machine learning. The SVM algorithm can construct a hyper-plane or set of hyper-planes in transformed input space to divide different classes and a good classification performance can be achieved by choosing the hyper-plane which has the largest distance to the closest data points of any class. To perform non-linear classification, the radial basis function was chosen as the kernel function.

### Random forest algorithm

Random forest algorithm is an ensemble learning algorithm using a decision tree as the base learner proposed by Breiman [26]. It grows multiple decision trees by searching for the best feature among a random subset of features and merges their classification results to decide the final classification with the bagging method.

### XGBoost algorithm

XGBoost stands for “Extreme Gradient Boosting” which is a superior implementation of gradient boosted decision trees designed for speed and performance proposed by Chen and Guestrin [27]. The algorithm is optimized in the utilization of computations resources; thus, it has been widely used and well evaluated in machine learning competitions. As the original algorithm, the Gradient boosted decision trees algorithm is also an ensemble learning algorithm using a decision tree as the base learner, but it uses a boosting method in which each tree attempts to minimize the errors of the previous tree and decide final classification with different importance or weights [28].

### Evaluation metrics

All classifiers in this paper are designed as multi-classification tools, so commonly used binary classification error evaluation metrics are not applicable. Overall accuracy can well represent the classification accuracy and was applied by other researchers in DRGs classifier evaluation [20]. However, the sample sizes tend to be uneven across categories. Without adjustment on this unbalanced data set, the model is easy to favour the large category and abandon the small category (for example, the ratio of positive and negative samples is 1:9, directly predicting all negative, overall accuracy also has 90%. But the positive sample is completely “thrown away”). High overall classification accuracy does not necessarily mean excellent performance of the classifier. Finally, we have chosen the kappa coefficient to quantify the accuracy of the grouping. Kappa coefficient is a score that expresses the level of agreement between two annotators on a classification problem [29]. In this paper, two annotators represent the case reference labels and the results of the grouping. Kappa coefficient is defined as

2 $$K=({p}_{o}-{p}_{e})/(1-{p}_{e})$$

where ${p}_{o}$ is the empirical probability of agreement on the label assigned to any sample, and ${p}_{e}$ is the expected agreement when both annotators assign labels randomly. For a confusion matrix as shown in Table 5, ${p}_{o}$ and ${p}_{e}$ can be calculated as:

3 $${p}_{o}=\frac{\sum_{i=1}^{r}{x}_{ii}}{N}$$

4 $${p}_{e}=\frac{\sum_{i=1}^{r}{(x}_{i+}\times {x}_{+i})}{{N}^{2}}$$

where $N$ is the number of samples. $r$ is the number of classes. A higher kappa coefficient indicates stronger classification accuracy than a lower one, and it can be interpreted referring to the guidelines [30] in Table 6.

**Table 5:** Confusion matrix schematic

Class	C1	C2	…	SUM
C1	${x}_{11}$	${x}_{12}$		${x}_{1+}$
C2	${x}_{21}$	${x}_{22}$		${x}_{2+}$
…				
SUM	${x}_{+1}$	${x}_{+2}$		${x}_{++}$

**Table 6:** Guidelines for interpreting the relationship between the kappa coefficient and classification accuracy

Kappa coefficient	0.01–0.20	0.21–0.40	0.41–0.60	0.61–0.80	0.81–1.00
Agreement	Slight	Fair	Moderate	Substantial	Almost perfect or perfect

In the process of models training, a tenfold cross-validation method is used to avoid any overfitting caused by unbalanced sample splitting. In the tenfold cross-validation, the dataset is divided into ten complementary subsets called folds. Then nine folds are used to train the groupings and the remaining fold is used as a test set. The process is repeated ten times until every fold has been used as a test set [31]. The final classification accuracy is calculated by the averaging performance of the ten groupings on their associated test sets.

## Results

### Performance of groupings

We would like to show that the rule generation method and the multiclass classification method can design a new grouping with similar performance to the original expert-oriented grouping. We also compare the performance of the five classification algorithms when they are applied in the multiclass classification method to give a reference for algorithm choice. Dataset A is used to train and test the groupings. Table 7 summarises the performance of the grouping which is trained and tested by simulated cases. Especially, the kappa coefficients of the rule generation method measure the classification accuracy of ADRG labels rather than DRG labels. The rule generation method achieves an average kappa coefficient of 0.9995, which proves that we can learn almost all clinical decision-making rules from existing expert-based groupings. The machine-learning algorithm related to the decision tree performs better in the multiclass classification method with average kappa coefficients of more than 0.9. Because the traditional grouping is also a tree structure, some features cannot satisfy class conditional independence, which leads to the relatively poor performance of the naive Bayes algorithm. Meanwhile, on multidimensional large datasets, SVM consumes a lot of time and memory but fails to achieve an excellent performance.

**Table 7:** Kappa coefficients of the data-based grouping trained by simulated cases

Folds	The rule generation method	Naive Bayes	SVM	CART	Random forest	XGBoost
1	0.9996	0.7141	0.7375	0.9218	0.9609	0.9680
2	0.9994	0.7104	0.7342	0.9221	0.9598	0.9710
3	0.9994	0.7067	0.7300	0.9218	0.9675	0.9688
4	0.9994	0.7069	0.7336	0.9192	0.9673	0.9712
5	0.9995	0.7101	0.7328	0.9197	0.9662	0.9669
6	0.9996	0.7064	0.7314	0.9216	0.9635	0.9709
7	0.9996	0.7126	0.7312	0.9210	0.9608	0.9676
8	0.9995	0.7067	0.7308	0.9202	0.9608	0.9719
9	0.9996	0.7067	0.7303	0.9210	0.9594	0.9703
10	0.9994	0.7120	0.7362	0.9207	0.9643	0.9680
Average	0.9995	0.7093	0.7328	0.9209	0.9631	0.9695

To verify that the grouping trained by simulated data could also be used in real data, Dataset B is used to test the groupings trained by Dataset A. To evaluate the effectiveness of the data-based update approach, the grouping with the best classification performance in the last experiment is selected to be the original grouping. The performance of groupings that are trained by simulated cases and tested by real cases is displayed in Table 8. Compared with being tested in the simulated cases, the groupings tested by the real cases have less accuracy, but the kappa coefficients of CART, random forest, and XGBoost algorithms are still higher than 0.6, which means that the results of these three groupings are still of the reference value and that these algorithms show good generalisation ability. Thus, designing grouping by simulated cases is a feasible idea when access to many real cases is severely restricted due to concerns about citizen privacy.

**Table 8:** Kappa coefficients of the data-based grouping trained by simulated cases

	*Naive Bayes*	SVM	*CART*	*Random forest*	*XGBoost*
Kappa coefficient	0.4274	0.3509	0.6454	0.6401	0.6803

In both experiments, the ensemble learning algorithm has a higher classification accuracy than the single decision-tree algorithm, but this improvement is not obvious. In the case of huge data and a lack of computation resources, a single decision-tree algorithm is still a good choice.

### Grouping update validation

Dataset C is divided like the tenfold cross-validation, and ten pairs of comparison data can be collected by testing every fold through the original grouping and the new grouping updated by the other nine folds. The performance comparison of the original grouping and updated grouping is shown in Table 9. The original grouping could not work accurately in the relabelled cases, which indicates that the effective decision-making rules for Dataset C are quite different from the rules in the CN-DRGs-B grouping. After being updated with a few relabelled cases, the average kappa coefficients of the grouping are significantly improved, from 0.1534 to 0.6435. Compared with the expert-oriented update, with enough computational resources, the update process can be completed in a very short time.

**Table 9:** Kappa coefficients of the original grouping and updated grouping

Folds	Original grouping	Updated grouping
1	0.1536	0.6167
2	0.1639	0.6691
3	0.1736	0.5877
4	0.1851	0.6556
5	0.1766	0.7090
6	0.1577	0.7304
7	0.1648	0.6850
8	0.1374	0.5874
9	0.0836	0.5863
10	0.1379	0.6074
Average	0.1534	0.6435

## Discussion

A data-based grouping is proposed in this study. We apply machine-learning algorithms to replace the non-professional work of experts in the process of grouping so that the grouping can be designed and updated based on data. Other research using machine-learning algorithms in DRG grouping [15, 18, 20] has focused on improving the performance of groupings or assisting hospitals in allocating medical resources, while we manage to prove that data-based grouping is feasible and superior to expert-based grouping.

Unlike traditional expert-oriented grouping, which is a black box, the design method and clinical-related rules of data-based grouping are disclosed. In addition, the rules of the non-public part of the data-based grouping are also dependent on the cases rather than on healthcare providers. Thus, this grouping could well avoid the problem of upcoding [32, 33]. The new method of grouping also gets rid of the dependence on experts, so we can use the cases whose labels are revised based on the actual local medical costs to update the original results of groupings. When the computation resources are sufficient, the groupings can be updated quickly at a low cost. When data-based groupings run in hospitals, with continuous feedback updates, the data-based grouping will correct the errors of expert-oriented groupings and grow with the advancement of healthcare reform and medical technology, thus providing the government and hospitals with real-time, localised management tools.

Since it involves patient privacy, it is very difficult for researchers to obtain enough real cases to run a study. This paper proposes a method to replace real cases with simulated cases that are generated by referring to the feature distribution of real cases. The groupings trained by the simulated data still have a good performance on the real data test set, so in the study of applying machine learning to DRG grouping, simulated data can help researchers verify their assumptions on the use and optimisation of algorithms in the exploration phase. In order for a data-based grouping to be well supervised and understood, simulated data preserve the interface of expert participation and ensure good interpretability by generating a rule tree and taking the OVA strategy.

We also compare five typical machine-learning classification algorithms. Based on the experimental results, we recommend that users choose the CART algorithm as the core of the multiclass classification method when computation resources are tight, and choose XGBoost when they are not.

## Conclusion

In this paper, we propose a data-based grouping based on machine learning, and completely demonstrate its design and update method. Through experiments, a data-based grouping was verified that meets the requirements of the DRGs system and has the advantages of high transparency and low cost in the design and update process. Hence, compared with the export-oriented grouping, the data-based grouping provides a more transparent potential option.

In the future, we will communicate with the government to obtain more real case data for training and improving groupings, while ensuring patient privacy. More experts will be invited to evaluate the problem of primary data, who will identify upcoding and the accuracy of diagnosis as well. We will also test our proposed grouping in cooperative hospitals, and improve our grouping according to feedback and suggestions from hospitals over a longer period, which also encourages health policy innovation [34].

## Data Availability

The datasets analysed during the current study are not publicly available due to involving personal information but are available from the corresponding author on reasonable request.

## Abbreviations

DRGs: Diagnosis-related groups
FFS: Fee-for-service
MDCs: Major diagnostic categories
ADRGs: The core diagnosis-related groups
SVM: Support vector machines
CART: Classification and regression trees
XGBoost: Extreme gradient boosting
OVA: One-vs-all
ICD: International classification of diseases
